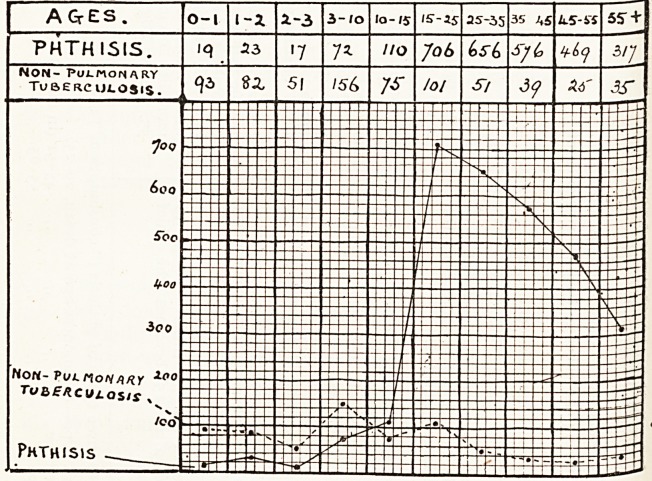# Tuberculosis and Consumption in Relation to Public Health

**Published:** 1920-12

**Authors:** D. S. Davies

**Affiliations:** Medical Officer of Health for the City and County of Bristol


### TUBERCULOSIS AND CONSUMPTION IN RELATION TO PUBLIC HEALTH.

BY

D. S. Davies, M.D. Lond., LL.D., D.P.H.,

*Medical Officer of Health for the City and County of Bristol.*

Tuberculosis in its various forms is the commonest of all the important diseases of civilised lands. It accounts for some 50,000 deaths a year in England and Wales; that is to say, about one death in every ten is due to tuberculosis.

The popular idea is that a young adult who develops pulmonary consumption has of necessity *recently* been infected by a relative, a companion, or a fellow-workman, just as happens in the case of scarlet fever, and much of the machinery in connection with " Sanatorium Benefit " seems to be frankly based on this assumption.

In considering the question of prevention it must be remembered that in the majority of civilised communities tuberculosis is as widely distributed and as constantly prevalent as the " common cold." Despite the fact that the new-born infant is practically always free from infection, hardly anyone who has attained the age of 14 has escaped.

**Distinction between Tuberculisation and Consumption.**—The distinguishing peculiarity about childhood infection is that as a general rule between the ages of 3 and 12 it does not manifest itself as consumption of the lungs, but may consist in a comparatively trivial affection of certain glands, prone to heal without further trouble, or in other cases may lead to more serious bone or joint or abdominal trouble, still curable if properly and early taken in hand. These *non-pulmonary* forms of tuberculosis, though they may prove fatal, are far less so than either the acute forms of generalised tuberculosis, which are terribly fatal during the first two years of life, or than the chronic pulmonary forms (consumption) which after the age of puberty cause most of the deaths from tuberculosis.

An important point about the practically universal childhood infection is that the severity of the case appears to depend, other things being equal, largely upon the " dose " received. If the dose is quite small (minimal dose) the disease is often so benign that it passes unnoticed, but it may end in a latent tuberculosis (the bacilli still alive but enclosed) of one or more glands. This bacillary infection with tuberculosis (tuberculisation), not "consumption," is compatible in the great majority of subjects with every appearance of health. This difference between "tuberculisation" and "consumption" is very important to bear in mind.

**"Immunity" in Tuberculosis.**—Childhood infection may confer a relative not absolute form of immunity. It affords fairly complete protection against acute, rapidly fatal forms in later life, but not complete protection against slower, chronic forms, which, while the patient is often well capable of holding his own, are yet sufficiently fatal; and these chronic patients are long standing and potent disseminators of infection.

These points already suggest very forcibly that it is too late if we only concentrate on even the earliest manifestations of chronic pulmonary phthisis (consumption); infection of another sort has already occurred years before. We must devote equal care to curing the slighter and benign manifestations in childhood in the hope that the maximum of possible "immunity" may be attained upon clinical recovery. Moreover, adults as well as children must be protected against exposure to "massive" infection, and against all depressing and predisposing causes, and if affected, then treatment with a view to cure must be taken in hand early. The anti-tuberculosis crusade must therefore be vigorously prosecuted against tuberculosis at all ages.

**Sources of Infection.**—The infection of tuberculosis may be received from other people, or from the cow through its milk. Generally speaking, the human source of infection is four times as important, even towards children, while chronic pulmonary phthisis (consumption) is practically always due to infection of human source. A large share of human tuberculosis is acquired during childhood from relatives in the intimate conditions of family life, the disease thus acquired not manifesting itself clinically (as consumption) for ten or twenty years afterwards, therein resembling the tertiary manifestations, after long latency, of syphilis.

**Influence of "Insanitary" Conditions.**—While it is correct to look upon overcrowding in cities or in rooms, bad ventilation, absence of sunlight, underfeeding, mental anxiety, and "insanitary" conditions or insanitary habits generally as powerful predisposing causes helping towards the wide diffusion and persistence of the disease, the essential factors are the persons or animals actually suffering, who distribute the infective germs. "Family" infection is a far greater danger, and leads to greater risk of "massive" infection, than does "bovine" infection. But although tuberculosis derived from the cow appears to be responsible for only 6 per cent. of the total deaths from tuberculosis, yet as this means 3,000 deaths in a year out of a total of some 50,000, it is of distinct importance; and it is inadmissible to contend that as bovine infection often leads to benign attacks, which appear to be protective, this source of risk may be neglected.

The fallacy of the contention consists in the fact that there is no control over the dosage received from cows' milk, and the dose, if massive, may readily lead to serious and prolonged illness. Every reasonable precaution should be taken to secure a milk supply as free from tubercle as possible, though even if this source of infection were entirely removed the far larger "human" risk still remains.

It is somewhat the fashion to say "tuberculosis is a question of housing." This seems to be an exaggeration, and hardly a useful one, as it tends to confusion between actual and predisposing causes, and distracts attention from the plain duty of exhausting or rendering harmless the actual sources of infection.

"No child will be found, were it condemned to live in the most unhealthy hovel under the most terrible conditions of want, nor any calf in the most insalubrious stable—which will contract tuberculosis, if in that hovel or in that stable virulent germs are not introduced, either intermittently or continually, by men or animals who are sick" with tubercle (Calmette).

Similarly alcoholism, poverty, malnutrition, unhealthy dwellings will not make a man tuberculous where the bacillus does not exist. But once the bacillus is present, these conditions paralyse or thwart the efforts of the natural defensive weapons, that is to say, act as most powerful predisposing causes. Therefore drastic action to remove such conditions, and especially to check overcrowding, and to secure adequate provision of fresh air, forms a necessary background to any scheme for the control of tuberculosis.

**The Decline in Consumption.**—The remarkable decline in the mortality recorded from "consumption" from 1840 to the end of the century, during which time the figures fell from 475 to 130, can hardly be due to any special measures taken, since it began and continued before any administrative action was commenced towards its control, and while administrative action has increased, the rate of decline shows signs of diminishing. The bills of mortality for London over a period of 200 years suggest that it is probably part of a declining wave of long duration, assisted possibly by improved economic conditions during the Victorian period; but this wave may show a future tendency to rise.

**Natural Selection in Disease.**—Independently of this decline, however, there is evidence that civilised communities, amongst whom the disease is constantly present, do enjoy a comparative immunity when compared with "virgin" populations (*i.e.* populations amongst whom the disease is newly introduced) as evidenced by a less mortality. Individuals vary in resistance to certain diseases, and under the constant weeding-out by the ever-present disease of the less resistant, though absolute immunity is not secured, *the mean resistance of a race towards the particular disease is raised.* This is true of malaria in West Africa, and much the same result seems to have occurred amongst civilisations where tubercle is endemic. For example, in 1917–18 the average strength of the British troops (from a country where the disease is endemic) in France was over a million and a half, but there were only 2,881 cases of tuberculosis, with 165 deaths, or about 1 death in 9,000. In the same period the average strength of Cape Boys and Kaffirs (from a country free from the disease) was only about 11,000, but the deaths from tuberculosis numbered 182, or about 16 deaths in every 1,000. The Fijian labour unit had to be repatriated on account of tuberculosis, and in 1916 the incidence of tuberculosis among the Indian divisions in France was 27.4 per 1,000, as compared with 1.1 per 1,000 British troops.

It is highly probable that high or low powers of resistance are transmitted by inheritance, and this point deserves more serious attention than it has hitherto received, in particular the mating of highly susceptible couples should in every way be discouraged. Heredity is important in the whole field of etiology, and becomes specially important in phthisis, because that disease is one of the largest factors of the death rate.*

**Protection due to Minimal Attacks.**—The second kind of racial immunity due to the continued and universal presence

* Dr. Paul A. Lewis, Henry Phipps Institute, Philadelphia, U.S.A.: An initial difficulty in experimenting was the variable resistance to tuberculosis encountered in different families of the same animal species. In order to achieve a uniform standard he carried out experiments on guinea pigs. In the course of these it had been determined that variations in susceptibility to infection depended on the factor of heredity more than on any other single factor. Whereas the ancestry of the guinea pigs counted for 30 per cent. of the variation, the weight and rate of growth counted for no more than 7–10 per cent. of this variation.

of the disease is not dependent on heredity, but is due to minimal cured or latent infections during the less susceptible childhood period. It is difficult to explain otherwise the terrible mortality where tuberculosis is introduced into a "virgin" population.

An example of this is seen in the 10 per cent. of total deaths from tuberculosis in England, compared with the 28 per cent. which followed its introduction amongst the Red Indians.

**The Risk to Infancy.**—Available evidence tends to show that infection in infancy is usually accomplished within the family. Tuberculosis is extremely rare in infants who live in homes where there is no tuberculous member, whatever the condition of the home.

Though the child will probably be born tubercle-free, there is great risk to infants suckled by a phthisical mother, especially if in an advanced stage. The infant is apparently not infected through the mother's milk, but by the bacilli she scatters in coughing or speaking. Similarly other tuberculous persons coming into contact with a nursling are a source of danger.

Up to the present public attention has been mainly focused on sanatorium benefit, of which the outstanding features are as follows:—

(*a*) Segregation of so-called *early cases* of pulmonary phthisis (consumption) in sanatoria.

(*b*) Segregation of advanced or "late" cases in hospital (acute cases).

(*c*) Tuberculosis dispensary or out-patient work, with searching out and supervision of contacts.

(*d*) Provision for domiciliary or home treatment.

The would-be best friends of the sanatorium, claiming impossible results, have unwittingly been its worst enemies, for there has been no marked failure within the limits of the possible influence of such methods, which have in fact been only directed against certain late manifestations of a long-standing infection. Many years of added useful life may have been obtained for a large number of individuals who respond to treatment, and for whom suitable after-care or suitable employment can be found, though much remains to be secured in this direction.

The sanatorium and its after-care complement, useful and necessary as they are, form but a small part of a complete scheme for the control of tuberculosis, which is a far wider question than merely dealing with chronic pulmonary phthisis (consumption).

Increasing attention, interrupted by the War, is now properly being given to searching out and making adequate provision for the non-pulmonary tuberculous affections of children of school age, and this necessary provision comprises :—

1. Open-air schools.

2. Sanatoria for "pre-tuberculous" cases in children (glandular cases, etc.).

3. Provision for dealing with the more serious cases of surgical tuberculosis, bone and joint affections and spinal cases in special hospitals.

Not only are these conditions more curable, and up to a point *probably protective in after-life*, but if neglected may result in serious and crippling deformity.

There is every need, therefore, to push forward this important work, not in place of sanatoria for consumption, but as the necessary and indispensable antecedent.

### SUMMARY.

(*a*) Tuberculosis is so universally present, and the opportunities for infection so frequent in most civilised communities, that, though born free, practically no one escapes infection of some sort by the tubercle bacillus. By the age of 14 over 80 per cent. are infected.

(*b*) Infection by the tubercle bacillus does not always result in obvious illness. Under 3 years of age infection will probably result in acute and fatal illness. Between 3 and 12 infection, if in moderate dose, will probably result in mild glandular affection, readily curable, and acting as some protection against further infection ; if the dose is large, however, serious bone or joint trouble may result. If not cured to sterilisation, the patient becomes a " carrier," liable to re-infect himself.

(*c*) Consumption (chronic pulmonary phthisis), which is rare at school ages, is met with more frequently in later life. It is a chronic form of lung disease, milder than acute tuberculosis, and in most instances appears to be due, not to recent infection, but to a re-awakening of previous childhood infection which was not absolutely cured. It only occurs in persons previously " immunised." If such a patient had not previously been partly protected by childhood infection an acute, rapidly-fatal tuberculosis would probably occur, and in unprotected populations infection results in very heavy mortality from rapid generalised tuberculosis.

(*d*) Of the sources of infection the " human " source is by far the most dangerous, but infection from the cow, though generally milder, is responsible for some amount of disease ; and as the " dosage " is not under control (except through the uncertain safeguard of " mixed " milk from large herds, most of which are presumably healthy), safeguards are necessary.

(*e*) Infants are peculiarly susceptible to " home " infection where parents or relatives are affected. " Sanitary conditions " do not exert their full depressing influence until past middle age.

(*f*) Consumptives do not appear to be a great source of danger to other adults, who are probably to some extent protected, but they are a constant danger to and the chief source of infection towards children in the home circle. While it may be admitted that early slight infection may produce resistant individuals, such persons require guarding against " massive " infection, and against depressing pre-disposing causes, lest they reinfect themselves.

**The necessary Institutional Provision in a Scheme for dealing with Tuberculosis.**—The Health Committee of the Corporation have been considering ways and means by which their sanatorium and hospital provision for " consumption " might be supplemented by the necessary antecedent provision of beds for the non-pulmonary forms of tuberculosis in childhood. The result of their deliberations has been the adoption of a scheme by the City Council, in which the major part of the provision is local, but certain special treatment for surgical cases for which no sufficient local facilities were immediately available will be obtained at Lord Mayor Treloar's Cripples' Home at Alton.

A comprehensive scheme for tuberculosis involves the following considerations:—

A. Preliminary Considerations.

1. The question of the health of parents is distinctly important, and the mating of definite consumptives should be discouraged in every way.
2. Consumptive mothers should not suckle their infants, and other consumptives in the home or family are a special danger to infants, who, though born free from tuberculosis, are most ready to contract it in fatal form.

B. Institutional Provision.

| | Bristol Sanatoria and Hospital provision. Beds in being. | Total Beds Approved. |
|---|---|---|
| *Non-Pulmonary Tuberculosis.* | | |
| 3. Provision for "pre-tuberculous" conditions in children, comprising open-air schools and special sanatoria (malnutrition, glandular affections) .. .. .. | 36 | 100 |
| 4. Provision in special hospitals for bone and joint affections (surgical tuberculosis) in children.. .. | — | 60 |
| 5. Provision for surgical aid in adult cases .. .. .. .. | — | 6 |
| 6. Provision for other non-pulmonary forms of tuberculosis (genito-urinary cases, etc.) .. .. | — | 12 |
| *Pulmonary Tuberculosis.* | | |
| 7. Sanatoria for "early" cases .. | 103 | 110 |
| 8. Hospitals for "late" cases .. | 53 | 93 |
| 9. Dispensaries (Portland Square and Redcliffe Parade) .. .. | — | — |
| 10. Continuation after-care provision, farm colonies, etc. .. .. | — | — |
| Total | 192 | 381 |

Provision is included under B.3 for the somewhat rare cases in children of school age, 2 per cent. of whom are apt to develop acute pulmonary phthisis, indicating either an unfavourable variation from the mean resistance, or a mean resistance overbalanced by excessive dosage.

### APPENDIX A.

TABLE A. TUBERCULOSIS IN RELATION TO AGE.

(In a community having the tubercle bacillus ever present.)

| Morbidity (sickness). | Nature of affection at different ages. | Fatality (deaths). |
|---|---|---|
| The new-born infant is practically always free from tuberculosis. | During the first year of life the membranes of the brain and lining membrane of the abdomen are largely affected. | *Infancy.*<br>In the first year of life tuberculosis is very fatal, especially from 6th to 12th month. About 80 per cent. die of those attacked. |
| During the first year of life about 15 per cent.<br><br>During the first five years about 50 per cent.<br><br>By the age of 14 about 80 per cent.<br><br>} Are infected in some form. | During the first two years of life acute miliary tuberculosis (a generalised and rapidly fatal form) and tuberculosis of bones, joints, and glands are common.<br><br>Between the ages of 2 and 10 the milder forms of bone, joint, and gland tuberculosis are mostly found. At these ages chronic pulmonary tuberculosis (phthisis or consumption) is very rare. | *Childhood.*<br>The school ages (period 5–15) is the period when deaths from tuberculosis are least frequent, though "infection" of some sort is most frequent.<br><br>*Adolescence, Middle Life, and Old Age.*<br>After the 15th year the mortality from tuberculosis, chiefly "consumption" increases, rapidly at first, afterwards more slowly. The maximum mortality occurs between 35 and 55, then falling slowly.[1] |
| After 30 practically everyone has been infected in some form, once at least. | After the age of 10 "phthisis" begins to prevail, and after 15 becomes the chief form of disease. | |

[1] Brownlee differentiates three types of phthisis ("consumption"):—

1. The young adult type—commonest age at death, 20–25.
2. The middle age type ,, ,, ,, 45–51 (in males).
3. The old age type ,, ,, ,, 55–65.

### APPENDIX B.

This chart shows in graphic form the deaths, classified by ages, from pulmonary phthisis (consumption) and from other forms of tuberculosis, for the seven years 1913 to 1919 inclusive, in the city of Bristol. The special prevalence of the "young adult" type at present in the city is clearly indicated.

| AGES. | 0-1 | 1-2 | 2-3 | 3-10 | 10-15 | 15-25 | 25-35 | 35 45 | 45-55 | 55+ |
|---|---|---|---|---|---|---|---|---|---|---|
| PHTHISIS. | 19 | 23 | 17 | 72 | 110 | 706 | 656 | 576 | 469 | 317 |
| NON-PULMONARY TUBERCULOSIS. | 93 | 82 | 51 | 156 | 75 | 101 | 51 | 39 | 25 | 35 |

### APPENDIX C.

TABLE SHOWING CAUSATION-RATIO OF BOVINE TO HUMAN TUBERCULOSIS (COBBETT).

| | *Human.* | *Bovine.* |
|---|---|---|
| Pulmonary tuberculosis. | Practically always. | 1.7 per cent. (Griffiths). |
| Tuberculous meningitis. | 82 per cent. | 18 per cent. |
| General tuberculosis (without meningitis). | 84 per cent. | 16 per cent. |
| Primary abdominal tuberculous affection. | 49 per cent. | 51 per cent. (none amongst adults). |
| Tuberculosis of bronchial glands. | In the great majority of cases human. | — |
| Tuberculosis of cervical glands. | — | Under 16 years of age 55.5 per cent. for England, 71.4 per cent. for Scotland. |
| Tuberculosis of bones and joints. | In most countries largely due to tubercle bacilli of human origin. | Edinburgh and neighbourhood appear to form an exception. |

### Notes.

In Edinburgh tuberculosis of bovine origin is apparently particularly rife. Comparing Edinburgh with Vienna, McNeill found the incidence of *tuberculosis in children up to* 4 *years* higher in Edinburgh, which is suggestive of milk infection, but the mortality from *pulmonary phthisis* in Vienna is three times as high as that for Edinburgh.

The human and bovine type of bacillus are probably varieties of one species adapted to different environment, but this differentiation is by no means absolute. Generally speaking, the bovine type is less virulent to man than the human type. But although the bovine type is extremely rare in the chronic adult pulmonary phthisis (consumption) of man, it is relatively common in glandular or acute infections in children. At the same time Calmette points out that tuberculosis is very common in children in countries where bovine tuberculosis does not exist, and where children are never brought up on cows' milk.

### AUTHORITIES CONSULTED.

1. Louis Cobbett, *The Causes of Tuberculosis*, Cambridge University Press, 1917.
2. *Reports to the Local Government Board on Public Health and Medical Subjects* (New Series, No. 122), H.M. Stationery Office, 1918.
3. Fishberg, *Pulmonary Tuberculosis*, Lea and Febiger, New York, 1919.
4. Pottenger, *Clinical Tuberculosis*, Kimpton, 1917.
5. Lawrason Brown, *The Control and Eradication of Tuberculosis*, Ed. by Sutherland, 1911.
6. Karl Pearson, *The Fight against Tuberculosis and the Death-Rate from Phthisis*, 1911.
7. Newsholme, *The Prevention of Tuberculosis*, 1908.
8. Brownlee, *An Investigation into the Epidemiology of Phthisis*. Nat. Health Insurance, Medical Research Committee, Part 1 and Part 2 Special Report Series No. 18. *Ibid.*, Part 3 Special Report Series No. 46.
9. Calmette, *L'inféction bacillaire et la Tubérculose*, Masson et Cie, Paris, 1920.
10. Prof. Hans Much, "Child Tubercle," quoted by Christopherson, *Tubercle*, 1920, ii. 1.
11. Walker, *Hereditary Characters and their Modes of Transmission*, Arnold, 1910.
12. Greenwood, "Epidemiology of Pulmonary Tuberculosis," *Annual Report of Chief Medical Officer, Ministry of Health*, 1920.
13. Prof. S. Lyle Cummins, "Tuberculosis in Primitive Tribes, etc.," *Internat. J. Pub. Health*, 1920, i. 137.
14. *Brit. M. J.*, 1920, i. 906. Review and Leading Article, "Tuberculosis."
15. G. Archdall Reid, *The Laws of Heredity*, Methuen, 1910.
16. F. Klemperer, *Die Lungentuberkulose*, Urban & Schwarzenberg, Berlin, 1920.

## Figures and Tables

**Figure f1:**